# An Interpretable Omics-to-Image Transformer Framework for Cancer Prognosis Prediction

**DOI:** 10.34133/csbj.0069

**Published:** 2026-04-24

**Authors:** Yanping Jiang, Wenhao Sun, Tianjun Lan, Yunkai Wu, Chaobin Pan, Hui Tang, Hua Chai

**Affiliations:** ^1^ School of Mathematics, Foshan University, Foshan 528000, China.; ^2^ Department of Oral and Maxillofacial Surgery, Sun Yat-sen Memorial Hospital of Sun Yat-sen University, Guangzhou, China.; ^3^ Guangdong Provincial Key Laboratory of Malignant Tumor Epigenetics and Gene Regulation, Guangdong-Hong Kong Joint Laboratory for RNA Medicine, Medical Research Center, Sun Yat-sen Memorial Hospital, Sun Yat-sen University, Guangzhou, China.

## Abstract

**Objective:** Accurate cancer prognosis prediction is essential for personalized treatment planning and clinical decision-making. With the rapid development of high-throughput sequencing technologies, multi-omics data such as messenger RNA expression, microRNA profiles, and DNA methylation provide complementary molecular perspectives on tumor progression. However, the high dimensionality, heterogeneity, interpretability, and complex cross-modal dependencies of multi-omics data pose substantial challenges to existing survival prediction models. **Methods:** To address these challenges, we propose an adaptive Omics-to-Image Transformer framework for Cancer prognosis Evaluation (OTCE), which converts heterogeneous multi-omics data into unified pseudo-image representations and further integrates them into multichannel image representations, facilitating effective cross-modal feature learning. A parallel multiview deep neural network composed of multiple functional modules is designed to capture global distributional characteristics, local spatial patterns, and long-range cross-modal dependencies, respectively. **Results:** Extensive experiments on 6 cancer datasets demonstrate that OTCE consistently outperforms state-of-the-art traditional and deep-learning-based survival models, achieving an average improvement of 7.7% in concordance index (C-index). Furthermore, by integrating Shapley-additive-explanations-based feature attribution with differential expression analysis, OTCE identifies 7 prognostic candidate biomarkers in kidney renal clear cell carcinoma, whose biological relevance is further supported by single-cell and spatial transcriptomic analyses. **Conclusion:** These results indicate that OTCE effectively improves the accuracy and robustness of multi-omics cancer prognosis prediction while enhancing model interpretability. The proposed framework provides a scalable and biologically meaningful solution for integrative survival analysis and offers valuable insights for prognostic biomarker discovery across multiple cancer types. The code of OTCE is available at https://github.com/fsct135/fsct135-2/tree/main.

## Graphical Abstract

**Figure:**
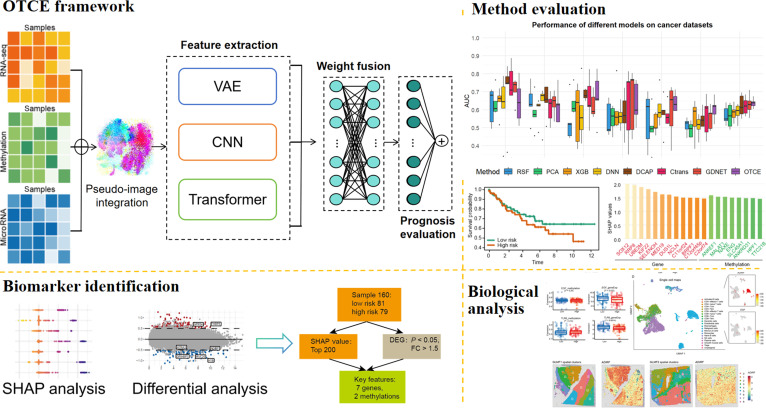

## Background

Clinical studies have demonstrated that the efficacy of therapeutic interventions is substantially compromised by the variability in outcomes observed among cancer patients who receive identical treatments. Accurate prediction of cancer prognosis is essential to inform subsequent therapeutic strategies, potentially prolonging survival and improving the quality of life [1,2]. With the advancement of molecular sequencing technologies, an increasing volume of high-dimensional omics data is now being leveraged through machine learning approaches to evaluate cancer prognosis and support clinical decision-making [3–5]. The messenger RNA (mRNA) data measured by microarray techniques [6,7] were widely used for prognosis evaluation, and with the development of next-generation sequencing techniques, many other types of data (microRNA [miRNA] [8], DNA methylation [9], etc.) are made available, which have great potential to overcome the inherent limitations of mRNA data and provide more comprehensive and in-depth views of cancer patients.

Integrating multi-omics data is advantageous in capturing the intricate nature of patients for disease research [10]. Different methods have been developed to integrate different omics data in many biological research areas [11–13]. Rohart et al. [14] developed a comprehensive software package based on sparse partial least squares–discriminant analysis for information extraction of multi-omics data. An unsupervised multiple kernel framework designed by Mariette and Villa-Vialaneix [15] was employed for the prediction of clinical outcomes in breast cancer. Kim et al. [16] proposed a grammatical evolution-based neural network for the assessment of ovarian cancer risks. A hierarchical clustering framework within a survival prediction algorithm developed by Tong et al. [17] was employed for the extraction of multi-omics data to predict colon cancer prognosis. Jardillier et al. [18] combined the proportional hazards model with a lasso-based regularization module for patient survival prediction. Additionally, considering the high-dimensional features of biodata, Widodo and Yang [19] applied a survival support vector machine with principal component analysis for proportional hazards evaluation.

In the past few years, deep learning (DL) techniques have demonstrated substantial advantages in effectively handling nonlinear features. DeepSurv is one of the advanced approaches to predict cancer prognosis that integrates a deep neural network (DNN) with the cancer survival evaluation task [20]. Cheerla and Gevaert [21] integrated different types of omics data into one DNN for cancer outcome prediction. Chaudhary et al. [22] utilized an autoencoder to extract representation of multi-omics features for liver cancer survival subtype identification. On this basis, Tong et al. [23] employed distinct autoencoders to model various omics features individually and subsequently integrated the generated features to prognosticate breast cancer outcomes. However, the segregation of feature extraction and prognosis analysis posed challenges to model training and testing. To solve this problem, a multitask DNN was designed to estimate patients’ risks by utilizing a loss function consisting of 2 modules: the data reconstruction module and the proportional hazards module [24]. In addition, considering that the application of these methods is still constrained by the limited availability of biodata samples, DNN frameworks based on meta-learning and contrastive learning strategies were used to predict prognostic risk in cancer patients, respectively [25–27]. However, these methods have proved to be of limited effectiveness in fusing complex multi-omics features, and there is still considerable scope for improvement in their performance in real-world data. Furthermore, the cancer analysis models constructed based on these DL methods lack feature interpretability, restricting researchers from gaining a deeper understanding of the key factors that influence the prognosis risk of patients.

Existing methods often model different omics modalities separately with limited cross-modal interaction and suffer from insufficient interpretability, restricting their biological and clinical applicability. To solve these challenges, in this study, we propose an adaptive Omics-to-image transformer framework for cancer prognosis Evaluation (OTCE). Specifically, OTCE employs multimodal correlation metrics to fuse different omics data in the form of pseudo-image representation, thereby facilitating cancer prognosis prediction by an explainable multiview parallel DNN. The main contributions of our research are given as follows:

• Multi-omics data are respectively transformed into 2-dimensional (2D) pseudo-images and further fused into 3-channel 3-dimensional image representations, enabling effective DL-based modeling of heterogeneous omics data.
• We design a parallel multiview DNN that incorporates different modules to extract global, local, and long-range cross-modal dependencies from the fused pseudo-images, enhancing the robustness and expressiveness of survival prediction.
• An adaptive attention-based fusion module is developed to dynamically integrate the complementary features extracted from different network branches, thus improving prediction performance across cancer types.
• OTCE was tested on different cancer datasets, and its prognosis prediction accuracy outperformed those of all compared methods by an average of >7.7% concordance index (C-index) values. The explainable result on a kidney renal clear cell carcinoma (KIRC) dataset shows a high correlation with tumor.

## Methods

### Datasets

In this study, the public cancer datasets used for method evaluation are collected from The Cancer Genome Atlas (TCGA) database (https://tcga-data.nci.nih.gov/tcga/) through the R package “TCGA-assembler 2” v1.0.3 [28]. Three types of omics data were utilized for integrative multi-omics analysis to predict cancer prognosis: mRNA, miRNA, and DNA methylation. Specifically, mRNA expression data were generated by the UNC Illumina HiSeq_RNASeq V2 platform, miRNA expression data were obtained from the BCGSC Illumina HiSeq miRNASeq platform, and DNA methylation data were generated using USC HumanMethylation450 BeadChip.

Since DNA methylation reflects site-specific information involving millions of variables, we extracted gene-level features by averaging the DNA methylation values across CpG sites within each gene as following a previous study [29]. Specifically, for each cancer type, features with missing values in more than 20% of the patients were removed. Subsequently, patient samples missing more than 20% of the remaining features were also excluded. We further excluded cancer types with fewer than 50 uncensored samples. For the remaining data, missing values were imputed using median values via the R package imputeMissings [30]. For the mRNA and miRNA data, the expression values were transformed through the log_2_ function. After preprocessing, all features were standardized to have a mean of 0 and a standard deviation of 1 across all cancer samples.

In addition, single-cell and spatial transcriptomes were used to validate the microenvironment mechanism of key features. The single-cell and spatial RNA sequencing data used in this study are publicly available in the Gene Expression Omnibus database at GSE210042 [31]. The Seurat R package (version 5.0.0) was utilized to process single-cell RNA sequencing and spatial transcriptomics data.

### OTCE architecture

**Fig. 1.**
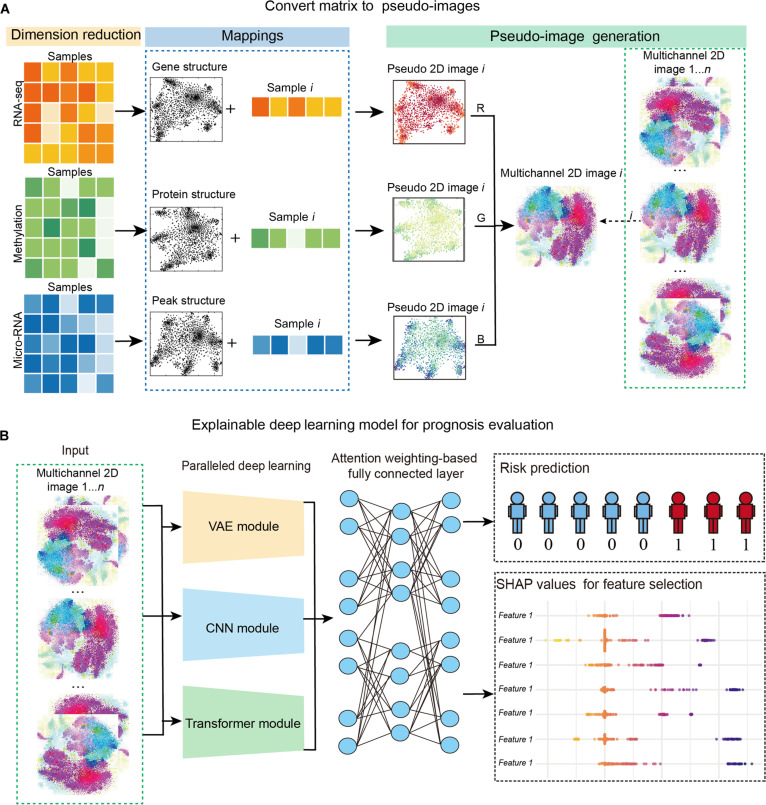
Schematic representation of the Omics-to-Image Transformer framework for Cancer prognosis Evaluation (OTCE) workflow. (A) Fundamental image structure constructed by transformation of multi-omics data from numeric vector to multichannel 2-dimensional (2D) image. First, multimodal data are processed via alternative feature extraction methods to obtain a 2D structure, and the molecule expression values of each cell are mapped to produce a 2D pseudo-image. Then, 2D pseudo-images of different modalities are combined to generate multichannel 2D images, which are the background data images and processed as the input of a deep learning network. (B) Explainable multiview parallel deep neural network for prognosis evolution and feature selection.

This study presents a multimodal data fusion DL framework for cancer prognosis risk prediction (Fig. 1). This approach integrates the features of mRNA, miRNA, and methylation data. The specific structural design is as follows: Firstly, the tabular data of the 3 modalities are respectively transformed into image forms to preserve the spatial distribution and global correlation of the data. This early fusion strategy allows the unified representation of heterogeneous modalities and avoids the limitations of traditional “separate modeling + late fusion” architectures, such as information loss and weak cross-modal interaction.

Subsequently, a multimodal parallel feature extraction network consisting of different function modules is constructed to conduct in-depth feature learning and representation optimization for each pseudo-image. The variational autoencoder (VAE) module is responsible for capturing the global distributional characteristics of the fused image by learning compact latent representations, which helps to uncover underlying prognostic structures. The convolutional neural network (CNN) module emphasizes local spatial variations and effectively detects localized expression patterns or hotspots within the multimodal image. Meanwhile, the transformer module, leveraging the self-attention mechanism, models long-range dependencies and intermodal correlations, allowing it to capture coordinated patterns across modalities and spatial regions.

The extracted multimodal features are dynamically weighted and integrated through an adaptive fusion module based on the attention mechanism to enhance the contribution expression of different modality features to cancer prognosis risk prediction. Eventually, the comprehensive features are input into the survival analysis network for the accurate prediction of patient prognosis risk.

### Constructing pseudo-image generation by multi-omics data fusion

Assume that we obtained multi-omics datasets $X_{i}\in {\mathbb{R}}^{m_{i}*n}$ from *n* number of patients with $m_{i}$ feature measured in different modalities (mRNA, miRNA, and DNA methylation). Following Tang et al.’s work [32], we reconstruct the features of each cell into a 2D grid of size that forms a 2D pseudo-image and then combine them from multiple modalities to generate a multichannel 2D image for each patient, which is the input of the OTCE. The details about components of pseudo-image generation are given as follows:

1. t-distributed stochastic neighbor embedding (t-SNE) [33] is used to transform the high-dimensional feature points in the observed data space to the visualizable deducted feature points in a latent space with 2 dimensions. The feature points of $X_{i}$ can also be represented as $X_{i}={\left(g_{1},g_{2},\ldots ,g_{m_{i}}\right)}^{T}$, where $g_{k}$ ($\begin{array}{c} k=1,2,\ldots ,m_{i} \end{array}$) is a vector with values of n patients. In this study, the feature matrix $X_{i}={\left(g_{1},g_{2},\ldots ,g_{m_{i}}\right)}^{T}$ is transformed by t-SNE for obtaining the 2D coordinates $\left\{\left(a_{1},b_{1}\right),\ldots ,\left(a_{j},b_{j}\right),\ldots ,\left(a_{m_{i}},b_{m_{i}}\right)\right\}$ of gene features in different types of omics data, and the coordinates $\left(a_{j},b_{j}\right)$ in the 2D plane defining the location of gene features $g_{j}$ in latent space. Then, a rotation is performed by a convex hull algorithm to frame the pseudo-image (i.e., 2D plane structure) horizontally or vertically for input into the following CNN architecture [34,35], and the Cartesian coordinates are converted to pixels to determine the location of each feature in the pixel frame.
  In this step, t-SNE has been shown to maintain the high-dimensional topology of features in a 2D plane and preserve the local relationships among features according to the similarity between features in the original high-dimensional space [33], thus allowing the association structure of features in the 2D plane to be determined and analyzed in a learning manner.
2. The molecular values of each patient j within vector $x_{j}$ are mapped to the molecular pixel locations of molecules in the 2D plane as detailed pixel values of the corresponding modality. The color shades of a single layer of an image range from 0 to 1. In this work, the pixel frame size is set to 224 × 224 by default, and the default value of each pixel is set to 1. Each $x_{\mathrm{ij}}$ is normalized as follows:

$$\begin{array}{c} \text{Step}\;1:x_{\mathrm{ij}}=\text{log}\left(x_{\mathrm{ij}}+\left|\underset{\text{samples}}{\text{min}}x_{\mathrm{ij}}\right|+1\right) \end{array}$$ (1)

$$\text{Step}\;2:x_{\mathrm{ij}}=\frac{x_{\mathrm{ij}}}{M}$$ (2)

The minimum value $\underset{\text{samples}}{\text{min}}x_{\mathrm{ij}}$ is adjusted for each feature across samples, and the global maximum value M=maxD of the whole training data $X_{i}$ is used to further scale the feature values between 0 and 1. Therefore, 2D pseudo-image data $z_{j}$ for each vector $x_{j}$ in one modality of one sample *j* can be generated.

$$z_{\mathrm{ij}}=\psi \left(x_{\mathrm{ij}}\right)\;\left(i=1,\;2,\;3;j=1,\;2,\;3,\ldots ,\;n\right)$$ (3)

This normalization approach will retain the association topology of all features. Notably, if more than one feature exists at the same location in the pixel frame, then these features will be placed in the same location, and their average expression values will be given as the final value of this pixel.

3. We combine the 2D images $z_{\mathrm{ij}}$ of 3 modalities in each sample j to generate a multichannel 2D image:

$$Z_{j}=\cup \left(z_{1j},\;z_{2j},\;z_{3j}\right)\;\left(k=1,\;2,\;3,\;\ldots ,\;n\right)$$ (4)

where n patients can be measured by the transformed n images, which are directly plugged into the following DNN framework. It is worth noting that we can randomly select 2D pseudo-images to form 3D image data, which would provide a consistent image production when the multimodal data have fewer than 3 modalities. In each cross-validation fold, the t-SNE embedding and pixel coordinate construction were performed exclusively using the training subset. The resulting coordinate layout was then fixed and applied to the corresponding validation/test samples without refitting the embedding model. This fold-wise processing ensures that no information from validation or test samples was used during pseudo-image construction.

It should be noted that the pseudo-image constructed via dimensionality reduction represents a computational embedding rather than a biologically grounded spatial organization. While such approaches preserve local feature relationships and facilitate the learning of intermolecular associations, they may not accurately maintain global distances, and future work will explore distance-preserving or biologically informed embedding strategies.

### The multiview parallel DNN

Using the above multichannel 2D images $Z_{k}\in {\mathbb{R}}^{224\times 224\times 3}$ $\left(k=1,\;2,\;3,\ldots ,\;n\right)$ as input and the cancer prognosis $y_{k}$ as output, a multiview parallel DNN is constructed for evaluation model training. OTCE extracts the multi-omics features of cancer data in different ways through 3 parallel modules, thus providing a more comprehensive patient profile. The fused multi-omics pseudo-images are processed in parallel by the 3 different modules, and complementary features are extracted from global, local, and cross-modal perspectives, thereby enhancing the model’s representational capacity and robustness.

The principal objective of VAE is to capture the latent structure distribution of the data, thereby extracting global features and offering stable and generative representations for multimodal feature fusion. VAE is composed of 2 core components: the encoder and the decoder. Given the input image data *X*, the encoder maps it into a latent space *z* while constraining the distribution of *z* to be close to the standard normal distribution. The decoder reconstructs the original data from the latent variables *z*:

$$z=\mu +\sigma ∙\epsilon ,\;\epsilon \sim N\left(0,1\right)$$ (5)

where $\mu =f_{\text{encoder}}\left(X\right)$, σ are the standard deviation of the latent space learned by the encoder network, and the reconstructed $X^{\prime }$ can be expressed as $X^{\prime }=f_{\text{decoder}}\left(z\right)$. The loss function of the VAE module is given as

$$L_{\mathrm{VAE}}=L_{\text{recon}}+L_{\mathrm{KL}}$$ (6)

where $L_{\text{recon}}={\left\|X-X\prime \right\|}^{2}$ and $L_{\mathrm{KL}}=D_{\mathrm{KL}}(q\left(z|X\right)|\left|N\left(0,1\right)\right)$, with $q\left(z|X\right)$ representing the potential variable distribution of the encoder network output. Here, μ is used as the compressed features generated by the VAE module: $F_{\mathrm{VAE}}=\mu$.

The module of CNN for local spatial features’ extraction in OTCE is composed of a convolutional layer, a pooling layer, and a fully connected layer. The features generated by the CNN module can be given as

$$F_{\mathrm{CNN}}^{l}=\text{Relu}\left(W^{l}*F_{\mathrm{CNN}}^{l-1}+b^{l}\right)$$ (7)

where $W^{l}$ and $b^{l}$ are the convolutional kernels and bias parameters of the *l* layer in the CNN module, respectively.

The transformer module is used to model the global structure of image features through self-attention mechanisms, capturing coordinated patterns across modalities and spatial regions, and extracting deep latent features. The weighted feature matrix of the single-head attention mechanism in the transformer module can be expressed as

$$X_{\text{attn}}^{i}=\text{Attention}\left(Q,\;K,\;V\right)=\text{softmax}\left(\frac{QK^{⊺}}{\sqrt{d_{k}}}\right)V$$ (8)

where *Q*, *K*, and *V* are the query, key, and value matrices, respectively. $d_{k}$ is the dimension of the attention head. The output features in transformer module can be given as

$$F_{\text{Trans}}=\text{Concat}\left(X_{\text{attn}}^{1},\;X_{\text{attn}}^{2},\;\ldots ,\;X_{\text{attn}}^{h}\right)W_{o}$$ (9)

where *h* is the number of attention heads and $W_{o}$ is the weight matrix.

To achieve adaptive fusion of different modal features, a dynamic weight allocation module based on the attention mechanism was added in OTCE:

$$F_{\text{fusion}}=\sum_{i=1}^{M}\alpha_{i}F_{i},\;\alpha_{i}=\frac{\text{exp}\left(h_{i}\right)}{\sum_{i=1}^{M}\text{exp}\left(h_{j}\right)}$$ (10)

where $F_{i}$ represents features generated by different function modules, $\alpha_{i}$ represents the attention weight, and $h_{i}$ represents the weight of feature $F_{i}$, generated by a fully connected layer.

The fusion feature $F_{\text{fusion}}$ is used to predict patients’ prognostic risk. The risk prediction follows the assumptions of the Cox proportional risk model:

$$h\left(t|x\right)=h_{0}\left(t\right)\text{exp}\left(w^{⊺}F_{\text{fusion}}\right)$$ (11)

where $h\left(t|x\right)$ represents the individual conditional risk function, $h_{0}\left(t\right)$ is the baseline risk function, and *w* is the model weight. Hence, the loss function can be expressed as

$$L_{\mathrm{Cox}}=-\sum_{i\in \text{uncensored}}\left(w^{⊺}F_{\text{fusion}}^{i}-\text{log}\sum_{j\in R_{i}}\text{exp}\left(w^{⊺}F_{\text{fusion}}^{j}\right)\right)$$ (12)

In this study, the Cox proportional hazards formulation is adopted as a partial-likelihood-based optimization objective. The DNN learns a nonlinear risk representation from pseudo-image inputs, and the Cox loss is used to preserve relative risk ordering among patients. The total loss function in OTCE is given as follows:

$$L_{\text{OTCE}}=L_{\mathrm{VAE}}+L_{\mathrm{Cox}}=L_{\text{recon}}+L_{\mathrm{KL}}+L_{\mathrm{Cox}}$$ (13)

By the parallel module in the DNN structure, OTCE can extract the patients’ information from different views. VAE captures the global distributional characteristics of the fused image, CNN pays attention to the details of local details, and the transformer excels in capturing coordinated patterns across modalities and spatial regions. This complementary nature enhances the model’s capability of depicting complex modal data and resisting noise.

### SHAP analysis

SHAP (Shapley additive explanations) values are computed at the pixel level based on the pseudo-images and the output predictions. Specifically, each pixel in the transformed omics-to-image representation is treated as an independent feature contributor during SHAP computation. The contribution of pixel i to the model prediction is computed as its Shapley value:

$$\emptyset_{i}=\sum_{S\subseteq N\backslash \left\{i\right\}}\frac{\left|S\right|!\left(n-\left|S\right|-1\right)!}{n!}*\left[f\left(S\cup \left\{i\right\}\right)-f\left(S\right)\right]$$ (14)

where N denotes the full set of pixels, S represents a subset of pixels excluding pixel i, n is the total number of pixels, and $f\left(S\right)$ is the model prediction when only the feature subset S is present. This formulation ensures that each pixel’s attribution reflects both its individual effect and its synergistic interactions with other pixels. After computing pixel-level SHAP values, each pixel is mapped back to its corresponding omics feature according to the deterministic encoding scheme used in the omics-to-image transformation. Gene-level importance scores are then obtained by aggregating absolute SHAP values across samples for pixels corresponding to the same feature, thereby preserving biological interpretability. SHAP values explain the impact of each feature (gene) on the model’s output, providing insights into gene importance and interactions. We applied SHAP using the SHAP Python package on all genes. To improve the reliability of feature interpretation under a limited sample size, the final model was trained on the full dataset using hyperparameters selected via cross-validation, and SHAP analysis was performed on this model. SHAP automatically identified gene interactions and interpreted the mean contributions of genes to risk prediction. We summed the SHAP scores for each gene across the sample to generate risk-level plots.

### Model evaluation

In cancer prognosis prediction, the estimation of prediction performance is commonly conducted using the C-index, which incorporates survival time as a factor. This approach is adopted due to the presence of numerous instances with uncensored survival time, which could potentially mislead predictors if directly used for survival time prediction. The C-index quantifies the proportion of correctly ordered predicted survival times among all pairs of individuals based on Harrell’s C statistics [36]:

$$C-\text{index}=\frac{\sum_{i=1}^{n}\sum_{j\neq i}\delta \left[\left(r_{i}-r_{j}\right)\left(y_{i}-y_{j}\right)\geq 0\right]}{\sum_{i=1}^{n}\sum_{j\neq i}\text{comp}\left(i,\;j\right)}$$ (15)

where r is the estimated prognosis situation by the model, y is the true survival time of the patient, and δ represents the censoring status of the sample. In addition, the area under the curve (AUC) of the time-dependent receiver operating characteristic curve is calculated for evaluating the model performance:

$$\mathrm{AUC}=\mathrm{Pr}\left(r_{i}>r_{j}|T_{i}>t,\;\;T_{j}\leq t,\;\delta_{j}=1\right)$$ (16)

where *t* is the survival interval of cancer patients.

### Parameter optimization and method comparison

In OTCE, 3 hyperparameters are used for model training. The parameter list for this study is as follows: The batch size was chosen from the set [32, 64, 128], the learning rate was chosen from the range [1e−3, 5e−4, 1e−4, 5e−5, 1e−5], and the number of fully connected lays was selected from 1 or 2. The number of epochs for training was 1,000. To further mitigate overfitting, several regularization strategies were implemented, including weight decay (1e−5), stochastic data augmentation (ColorJitter with *P* = 0.5), and selecting the parameters based on the 5-fold cross-validation results in the experiments. More details about the parameters are given in Table S2. Additionally, the learning rate [200, 500, 1,000] and perplexity [20, 30, 40] in t-SNE are treated as tunable hyperparameters during pseudo-image construction.

We compared the cancer prognosis evaluation performance obtained by OTCE with 3 traditional methods (random survival forest [RSF], Cox model with principal component analysis [PCA-Cox], and XGBoost) and 4 DL-based methods (DNN for survival analysis, DCAP [29], Cox model with transformer-based autoencoder [CTrans], and GDNet [27]).

### Biological significance analysis

Given the high degree of molecular heterogeneity and the challenges in prognostic stratification of cancers, there is an urgent need to identify stable and predictive molecular features. Therefore, this study selected KIRC as the primary focus for downstream analysis, aiming to further explore key features that have a critical impact on patient prognosis. To enhance the biological interpretability of the model’s predictions, we adopted a dual-perspective approach by integrating SHAP values and differential expression analysis (DEA) to evaluate the importance of gene features used in the model. SHAP was employed to quantify the contribution of each gene to the model’s predictions, while DEA was used to identify genes with significantly different expression levels between risk groups. The combination of these 2 methods facilitates the identification of key genes that are both statistically significant and functionally important within the model, thereby providing a more reliable basis for subsequent mechanistic studies and potential biomarker discovery.

## Results

### Patients’ risk estimation by multi-omics data

We sought to design a robust and flexible DL model for the integration of multiple measurements collected within the sample. To be applied to a wide range of biological data types, our methods must successfully address the following criteria: First, compared to existing integration methods, our approach can achieve a higher and robust accuracy. Second, our integration methods should obviously improve the ability to predict risk groups, compared to the independent analyses of each modality when performed separately. Last, the integration strategy should execute feature engineering, feature selection, and topic recommendation in the biological context. These challenges highlight the importance of a flexible model for handling multimodal datasets. Technically, the workflow of OTCE includes 3 steps: (a) constructing a tensor feature correlation/coordinate structure in a latent feature space using an alternative feature extraction method (t-SNE) to transform each modality data into a pseudo-image, which contains both feature abundance and correlation information (Fig. 1A); (b) processing the pseudo-images in 3 different parallel modules and subsequently integrating the extracted representations through an attention-weighted multilayer perceptron for risk prediction (Fig. 1B); and (c) incorporating SHAP value analysis for feature selection (Fig. 1B).

To evaluate the prognostic performance of the proposed model OTCE across multiple cancer types, we computed the C-index and AUC on 6 TCGA datasets: breast invasive carcinoma (BRCA), cervical squamous cell carcinoma and endocervical adenocarcinoma (CESC), colon adenocarcinoma (COAD), esophageal carcinoma (ESCA), KIRC, and lung squamous cell carcinoma (LUSC). For the C-index evaluation, the model achieved the best performance on the COAD (0.659) and KIRC (0.634) datasets, while relatively lower performance was observed on LUSC (0.594). The overall average C-index was 0.624, indicating reliable discrimination in survival risk prediction across different cancer types, and the average AUC across all cancer types reached 0.637 ± 0.022, further validating the generalization capability and robustness of the model in pan-cancer survival prediction tasks (Table 1).

**Table 1.** The cancer prognosis evaluation performance obtained by OTCE

Datasets	BRCA	CESC	COAD	ESCA	KIRC	LUSC	Average
C-index	0.624 (±0.026)	0.614 (±0.067)	0.659 (±0.103)	0.617 (±0.064)	0.634 (±0.026)	0.594 (±0.040)	0.624 (±0.020)
AUC	0.641 (±0.046)	0.618 (±0.086)	0.675 (±0.120)	0.636 (±0.102)	0.641 (±0.046)	0.605 (±0.055)	0.637 (±0.022)

OTCE, Omics-to-Image Transformer framework for Cancer prognosis Evaluation; BRCA, breast invasive carcinoma; CESC, cervical squamous cell carcinoma and endocervical adenocarcinoma; COAD, colon adenocarcinoma; ESCA, esophageal carcinoma; KIRC, kidney renal clear cell carcinoma; LUSC, lung squamous cell carcinoma; C-index, concordance index; AUC, area under the curve

### Method comparison

**Fig. 2.**
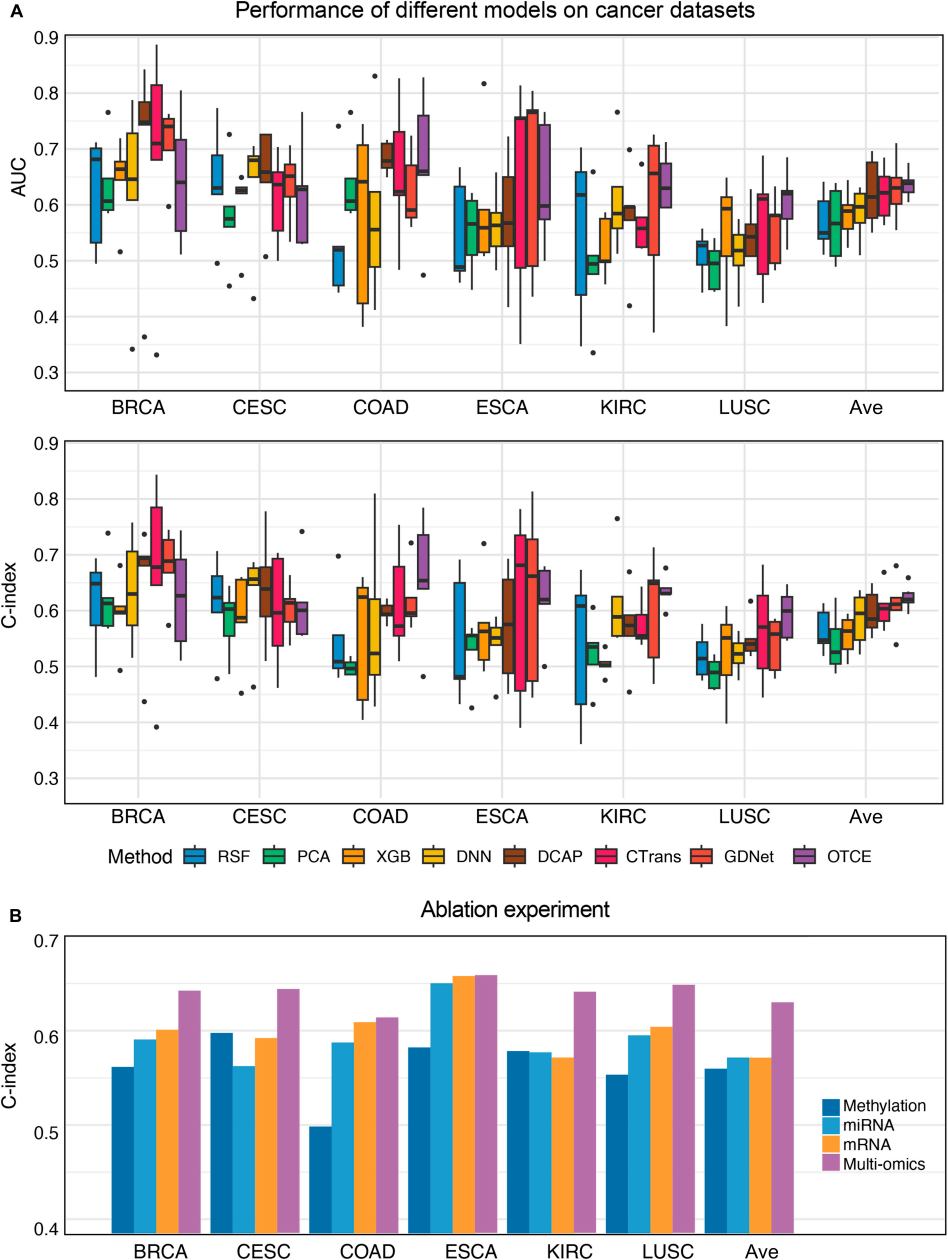
Performance evaluation and comparison. (A) Prediction performance of cancer outcomes, evaluated by area under the curve (AUC) and concordance index (C-index) in 5-fold cross-validation experiments, obtained using Omics-to-Image Transformer framework for Cancer prognosis Evaluation (OTCE) across 6 cancer types. (B) Benchmarking the performance of 5-fold cross-validation on single-modality datasets as the ablation experiment.

To comprehensively evaluate the prognosis evaluation performance of various survival analysis methods, we conducted 5-fold cross-validation experiments on 6 TCGA cancer datasets. Eight models were compared, namely, RSF, PCA-Cox, XGBoost, DNN, DCAP, CTrans, GDNet, and our proposed OTCE. We give their C-index values obtained by different methods across datasets in Fig. 2A. The PCA-Cox method achieved the lowest C-index values with an average of 0.540, and the 3 traditional methods (RSF, PCA-Cox, and XGBoost) achieved average C-index values of 0.553, which are lower than that of the DL-based methods (0.604). GDNet performed better than DNN, DCAP, and Ctrans but worse than our proposed OTCE. OTCE achieved the highest C-index values between 0.594 (LUSC) and 0.659 (COAD), with an average of 0.624. Compared with other methods, OTCE improved C-index values by 7.7% on average. To further evaluate the statistical significance and stability of model performance, paired *t* test results and 5-fold cross-validation standard deviations are provided in Table S1. Overall, these results indicate that the proposed OTCE method offers improved stability and prognostic accuracy across various cancer types, underscoring its effectiveness in multi-omics integration and survival prediction tasks.

To further assess the cancer prognosis performance of each method, the AUC is shown in Fig. 2A. Overall, our proposed OTCE model achieved the highest average AUC of 0.637, outperforming all baseline methods. It demonstrated leading performance in ESCA (0.636), CESC (0.618), COAD (0.675), and KIRC (0.641), indicating its superior capability in distinguishing different prognosis risk patient groups. GDNet ranked second with an average AUC of 0.629, showing excellent results in BRCA (0.710) and COAD (0.625), which highlights the advantage of deep neural architectures in modeling complex data distributions. DCAP and CTrans also performed competitively, suggesting their reliability across different types of cancer. In contrast, traditional models such as RSF and PCA-Cox showed relatively inferior performance, particularly in highly heterogeneous cancers like LUSC and KIRC. This reflects their limitations when handling multimodal omics data. When considering both C-index and AUC metrics, OTCE consistently outperforms state-of-the-art methods, demonstrating strong generalization and discriminative capability, which underscores its potential in multi-omics-based survival analysis for different cancers.

### Omics importance evaluation

To investigate the contribution of different omics modalities to cancer prognosis prediction, we constructed survival models based on individual omics data (mRNA expression, DNA methylation, and miRNA expression) as well as integrated multi-omics features. As shown in Fig. 2B, when using a single type of omics data, mRNA performed the best with an average C-index value of 0.601, and DNA methylation data had the lowest performance with a C-index value of 0.562. On average, the multi-omics model achieved a C-index of 0.624, which is notably higher than those of mRNA (0.601), methylation (0.562), and miRNA (0.591). The findings highlight the effectiveness of omics integration in enhancing the accuracy of survival outcome prediction.

### SHAP value investigation reveals complex gene behaviors and interactions

**Fig. 3.**
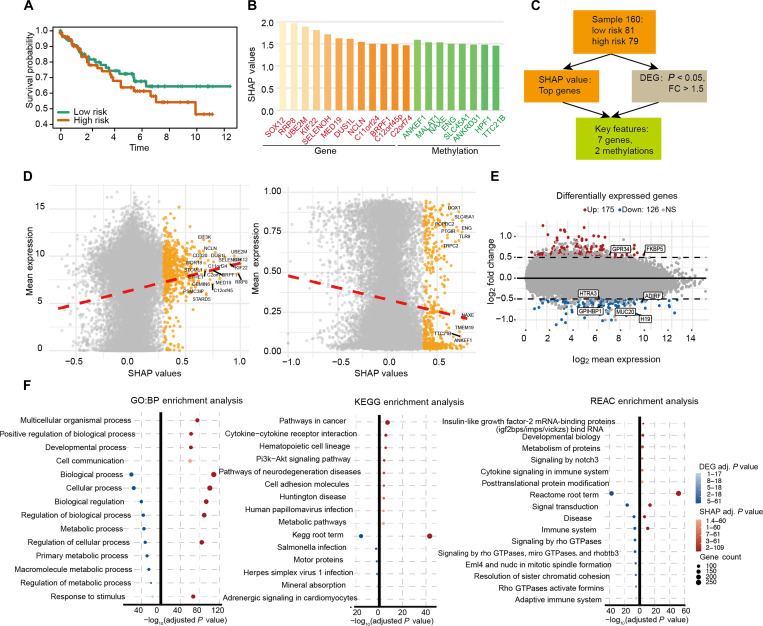
The case study on kidney cancer. (A) The Kaplan–Meier survival curves show the clinical relevance of the 2 risk groups. (B) Top 20 features with absolute Shapley additive explanations (SHAP) scores identified in the model. (C) Intersection plots illustrating the key features with higher SHAP values and significant differential expressed in 2 risk groups. (D) Correlation between SHAP values and molecular features. Each dot represents a gene or a methylation site, with gray indicating nonsignificant features and orange highlighting those with high SHAP contributions. The *y*-axis shows mean expression (for genes) or methylation level, while the *x*-axis shows SHAP values. The red dashed line denotes the fitted trend, and representative top-ranked genes are labeled. (E) The differentially expressed genes for risk groups identified by Limma in liver cancer. The blue nodes represent down-regulated risk genes, and the red nodes indicate up-regulated risk ones (|log_2_FoldChange| > 1.5 and corrected *P* value < 0.05). (F) The enrichment dot plot shows the significantly enriched pathways of informative genes (false discovery rate [FDR] < 0.05).

We next applied our approach to KIRC as the primary focus of downstream analysis, with the aim of further investigating gene features that critically influence patient prognosis. As shown in Fig. 3A, the high-risk and low-risk groups classified by OTCE can be significantly separated from the survival curves. In Fig. 3B, we present the top 20 features ranked by SHAP values, including 12 genes and 6 methylation sites. Several studies have reported that some of these genes, such as *SOX12* and *RRP8*, possess prognostic value in KIRC [37,38]. Although the association of *MALAT1* and *ANKEF1* methylation with KIRC has not yet been documented, it is noteworthy that *MALAT1* overexpression has been linked to tumor progression, enhanced invasiveness, and poor prognosis, while data from the Human Protein Atlas suggest that *ANKEF1* may serve as a potential prognostic marker in KIRC [39].

To enhance the biological interpretability of the model’s predictions, we adopted a dual-perspective approach by integrating SHAP values and DEA to evaluate the importance of gene features used in the model. To identify prognostic candidate biomarkers in KIRC, we intersected the top 200 genes ranked by SHAP values with differentially expressed genes (DEGs) (|logFC| > 0.5, *P* < 0.05) identified by DEA based on the survival analysis result obtained by OTCE (Fig. 3C). The results reveal that although most higher SHAP value features do not exhibit significant differences between the 2 risk groups, their consistently elevated expression suggests that they may play important biological roles and contribute to the prognostic relevance captured by the model (Fig. 3D and Fig. S1). The DEA was performed based on the 2 risk groups predicted by OTCE and identified 301 DEGs with *P* value < 0.05 and |log_2_ fold change| >0.5, among which there were 175 down-regulated risk and 126 up-regulated risk genes (Fig. 3E and Fig. S2). We performed functional enrichment analysis by integrating DEGs with the top 200 genes ranked by SHAP values. Kyoto Encyclopedia of Genes and Genomes enrichment analysis revealed that the associated pathways were predominantly cancer related, while Reactome analysis highlighted pathways mainly involving Notch3 signaling and immune-related processes (Fig. 3F). These findings further validate the biological relevance of OTCE in prognostic prediction.

### In-depth characterization of model-derived key features

**Fig. 4.**
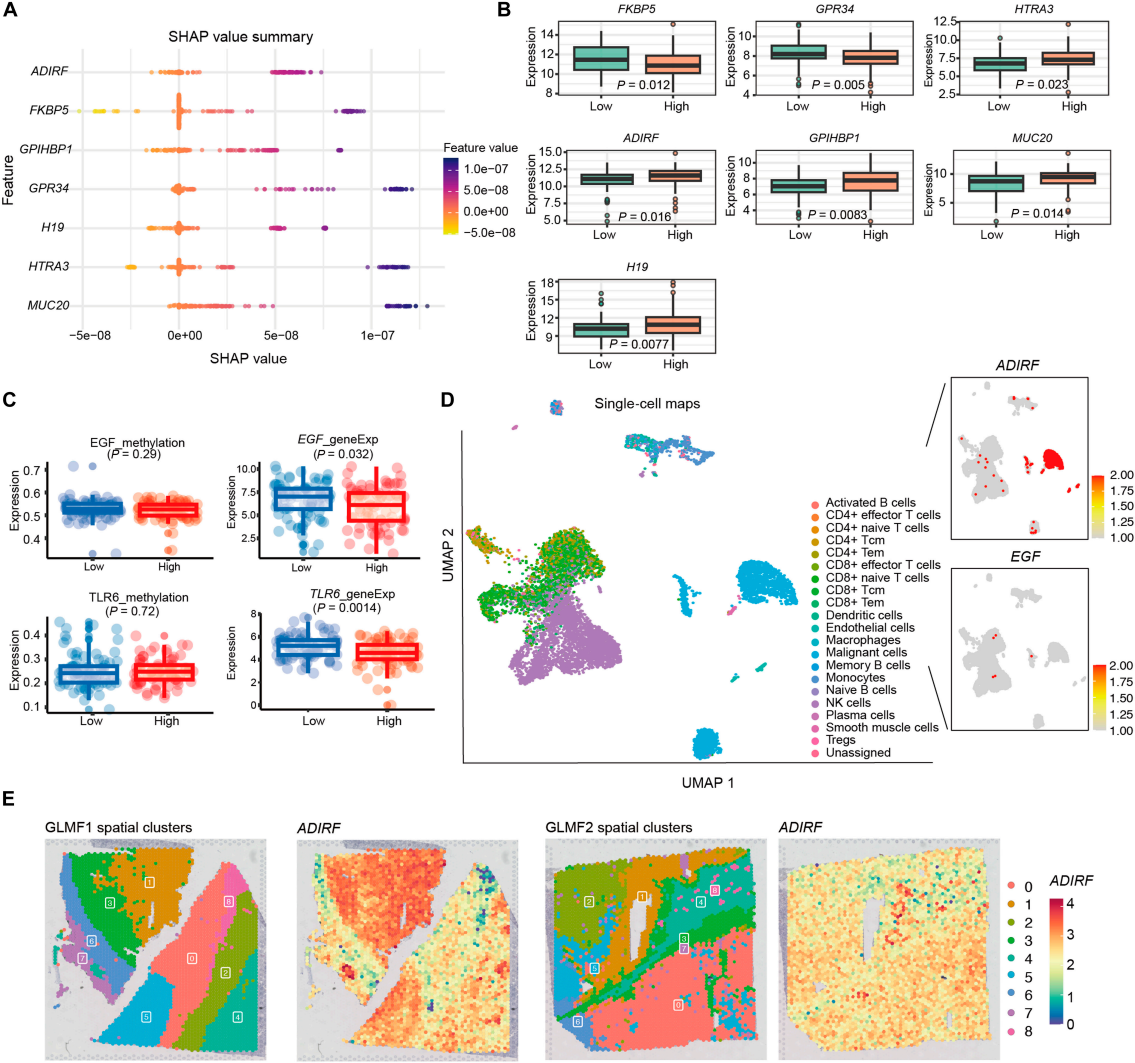
Deep analysis of key features. (A) Shapley additive explanations (SHAP) value summary plot depicting the impact of each of the most key genes on the model output. (B) Boxplots show the expression of key genes in 2 risk groups. (C) Boxplots show the distribution of methylation and expression for *EGF* (top) and *TLR6* (bottom). The *P* values indicate the statistical significance of differences between the 2 groups. (D) Single-cell uniform manifold approximation and projection (UMAP) visualization of different cell populations (left) and feature plots showing the expression of *ADIRF* and *EGF* (right). (E) Spatial plot showing the expression of *ADIRF*.

Seven overlapping DEGs were identified, namely, *MUC20*, *GPR34*, *ADIRF*, *HTRA3*, *GPIHBP1*, *H19*, and *FKBP5*, which may play pivotal roles in patient prognosis and tumor progression (Fig. S3). The SHAP scores of these 7 genes are shown in Fig. 4A, and the corresponding gene expression values are shown in Fig. 4B. In addition, 2 methylation sites, EGF and TLR6, were found among the top 200 SHAP-ranked features overlapping with DEGs. These sites exhibited reduced methylation, correlating with significant regulation of their respective gene expression, suggesting potential roles in tumor progression (Fig. 4C). According to National Center for Biotechnology Information Gene annotations (https://www.genecards.org/), *H19* is a long noncoding RNA which functions as a tumor suppressor [40]. *HTRA3* may inhibit signaling mediated by TGF-beta family proteins, possibly through the indirect degradation of extracellular matrix proteoglycans, which suggests its potential role as a tumor suppressor [41]. *ADIRF* plays a role in fat cell development, and its overexpression confers resistance to the anticancer chemotherapeutic drug cisplatin. *GPR34* has been reported as a metabolic immune checkpoint for ILC1-mediated antitumor immunity [42]. Moreover, *FKBP5*, *MUC20*, and *GPIHBP1* are also associated with the prognosis of other cancers. To further investigate the microenvironmental mechanisms associated with these genes, we collected single-cell and spatial transcriptomics data from KIRC. Analysis revealed that *ADIRF* was significantly enriched in cancer cells and regions (Fig. 4D and E and Figs. S4 and S5). Given its role in lipid metabolism and the lipid-rich phenotype of KIRC, *ADIRF* may contribute to tumor progression and microenvironmental interactions, supporting its potential as a prognostic marker. In addition, we performed independent Kaplan–Meier survival analyses for the 7 candidate genes identified by overlapping SHAP attribution and DEA in KIRC. The results show that 5 of the 7 genes exhibit statistically significant survival differences, with higher expression consistently associated with the high-risk group, supporting their prognostic relevance beyond model-derived importance (Fig. S6). These findings demonstrate that OTCE can construct a cancer prognosis evaluation model with enhanced biological interpretability, which is helpful for biomedical applications.

## Discussion

The proposed OTCE framework in this study demonstrated superior prognostic prediction performance. By transforming multi-omics data into a unified pseudo-image representation and applying different function modules in parallel, OTCE effectively captures global, local, and cross-modal dependencies, achieving enhanced feature fusion and representation. The experimental results confirm that the integration of mRNA, miRNA, and DNA methylation data leads to significant improvements in both C-index and AUC compared to single-modality models, supporting the efficacy of multi-omics integration for cancer prognosis modeling.

Despite these strengths, OTCE has certain limitations worth discussing. Firstly, the pseudo-image generation process relying on the dimensionality reduction technique may result in information loss from the high-dimensional space. Secondly, the censored and limited samples in the data restrict the accuracy of predicting cancer outcomes. Thirdly, the previous study has proved that integrating more different omics data, such as single-cell and image data, is helpful to improve the cancer prognosis prediction performance [43–45]. This is a potential way to improve model prediction performance.

In the future, we will optimize the nonlinear dimensionality reduction technique and incorporate uncertainty modeling techniques and semisupervised learning strategies to help mitigate the negative impact of small sample sizes and censored labels, thereby improving generalization and reliability. Furthermore, considering that the mapping from features to pseudo-image pixels may introduce approximation bias during feature aggregation, we will explore more credible and robust attribution methods such as the integration of SHAP and integrated gradient algorithm.

## Conclusion

In summary, we designed the adaptive omics-to-image transformer framework OTCE for cancer prognosis. This approach integrates 3 parallel DNN modules to conduct complementary feature learning on complex multi-omics data, fully exploring the characteristics of different modality data. OTCE also employs an adaptive fusion strategy based on the attention mechanism to dynamically optimize the weight distribution of multimodal features and effectively integrate cross-modal information. In this manner, OTCE effectively addresses the challenges associated with integrating heterogeneous omics data, thereby substantially improving the accuracy and reliability of cancer prognosis evaluation. By combining the results of DEA and SHAP, OTCE boosts the interpretability of the model, providing support for parsing key biological mechanisms. Additionally, this framework possesses excellent universality and scalability, being applicable to multiple cancer types and other omics data, offering an efficient technical solution for complex disease research.

## Data Availability

All data analyzed during the current study are available in the TCGA dataset: https://tcga-data.nci.nih.gov/tcga. The code of OTCE is available at https://github.com/fsct135/fsct135-2/tree/main.
