# Supplementary material for: An Interpretable Omics-to-Image Transformer Framework for Cancer Prognosis Prediction
Source: Comput Struct Biotechnol J. 2026 Apr 24;35(1):0069. doi: 10.34133/csbj.0069 (PMC13106938; doi:10.34133/csbj.0069)
Supplement: Supplementary 1 — Figs. S1 to S7 Tables S1 and S2 [file csbj.0069.f1.pdf]

An Interpretable Omics-to-Image Transformer Framework for Cancer Prognosis Prediction

Yanping Jiang[1#], Wenhao Sun[2#], Tianjun Lan[2,3], Yunkai Wu[1], Chaobin Pan[2,3], Hui Tang[1*], Hua Chai[1*]

[1]School of Mathematics, Foshan University, Foshan 528000, China.

[2]Department of Oral and Maxillofacial Surgery, Sun Yat-sen Memorial Hospital of Sun Yat-Sen University, Guangzhou, China,

[3]Guangdong Provincial Key Laboratory of Malignant Tumor Epigenetics and Gene Regulation, Guangdong-Hong Kong Joint Laboratory for RNA Medicine, Medical Research Center, Sun Yat-sen Memorial Hospital, Sun Yat-sen University, Guangzhou, China.

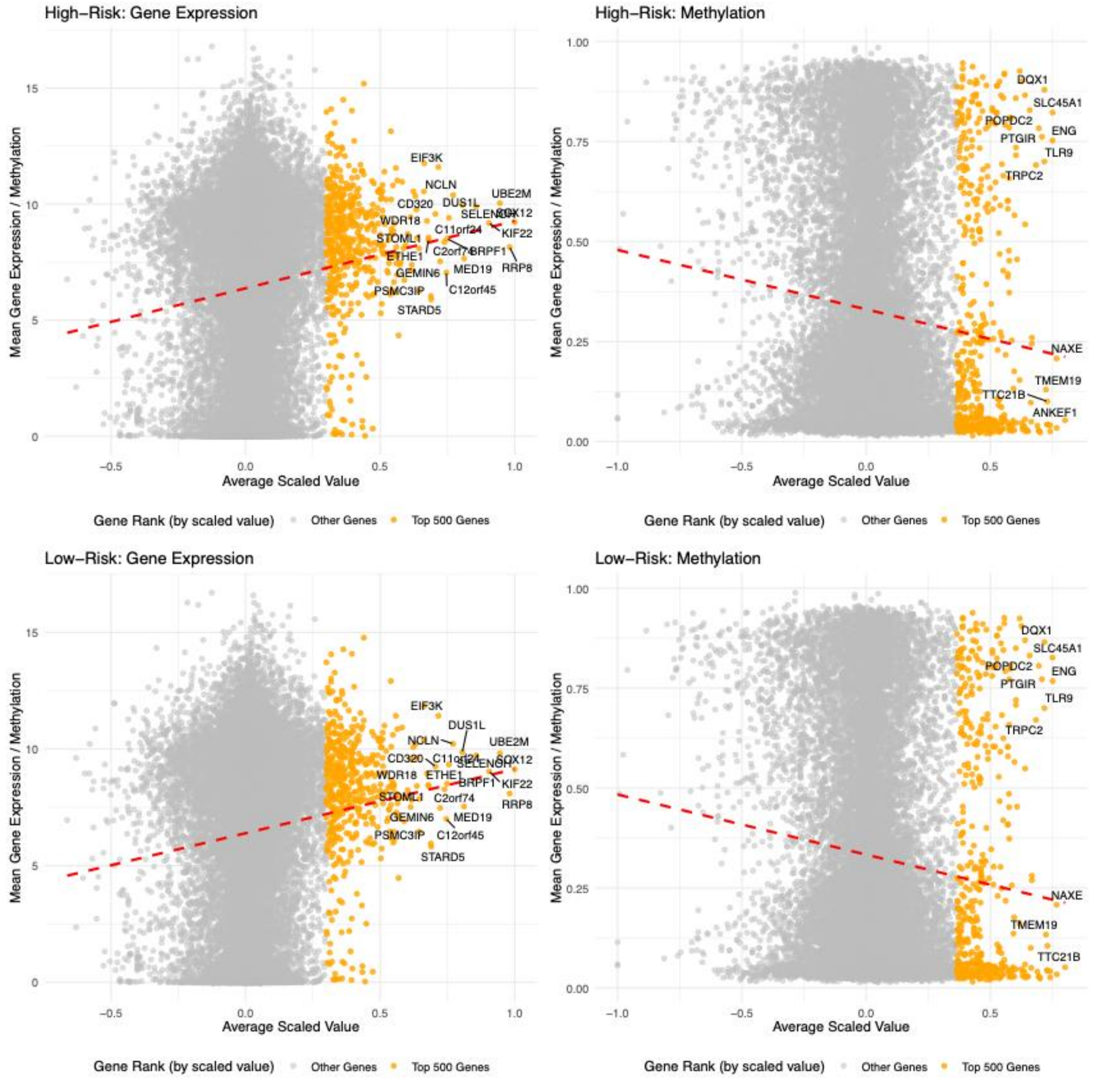

Fig. S1 Correlation between SHAP values and molecular features. Each dot represents a gene or methylation site, with gray indicating non-significant features and orange highlighting those with high SHAP contributions. The y-axis shows mean expression (for genes) or

methylation level, while the x-axis shows SHAP values. The red dashed line denotes the fitted trend, and representative top-ranked genes are labeled.

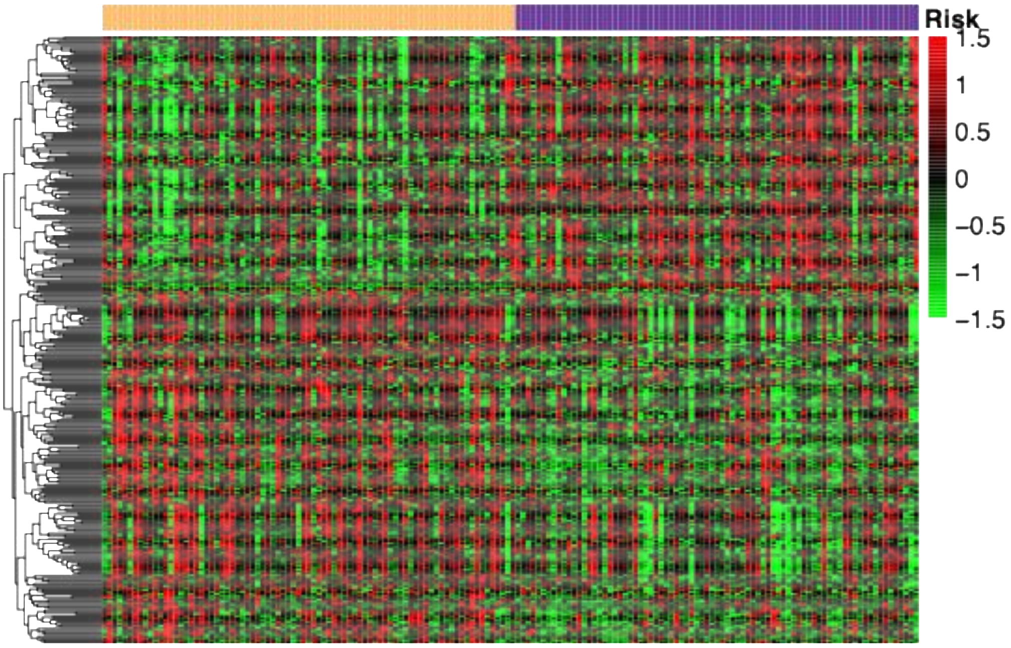

Fig. S2 The clustering heatmap of DEGs.

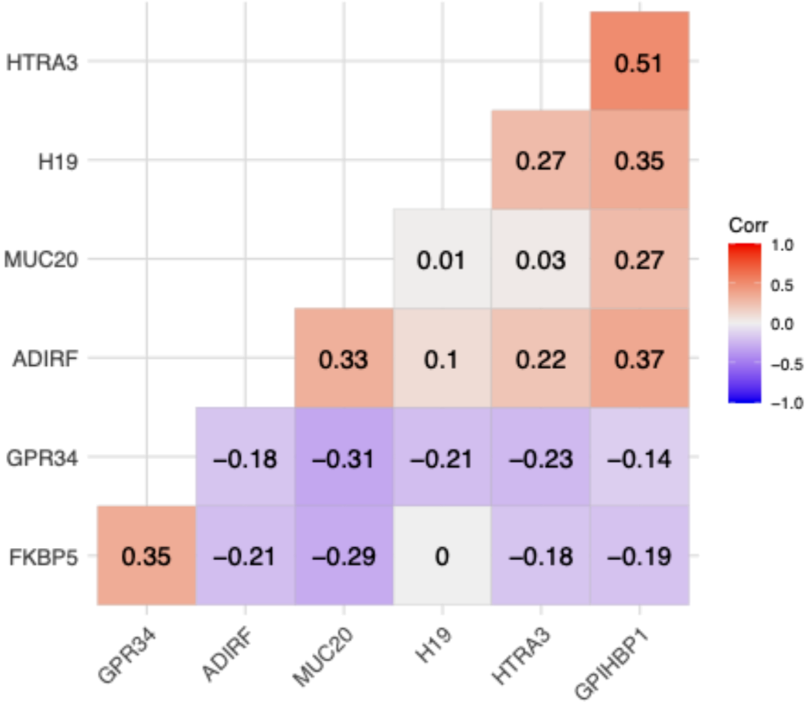

Fig. S3 Correlation analysis between the expression levels of key genes.

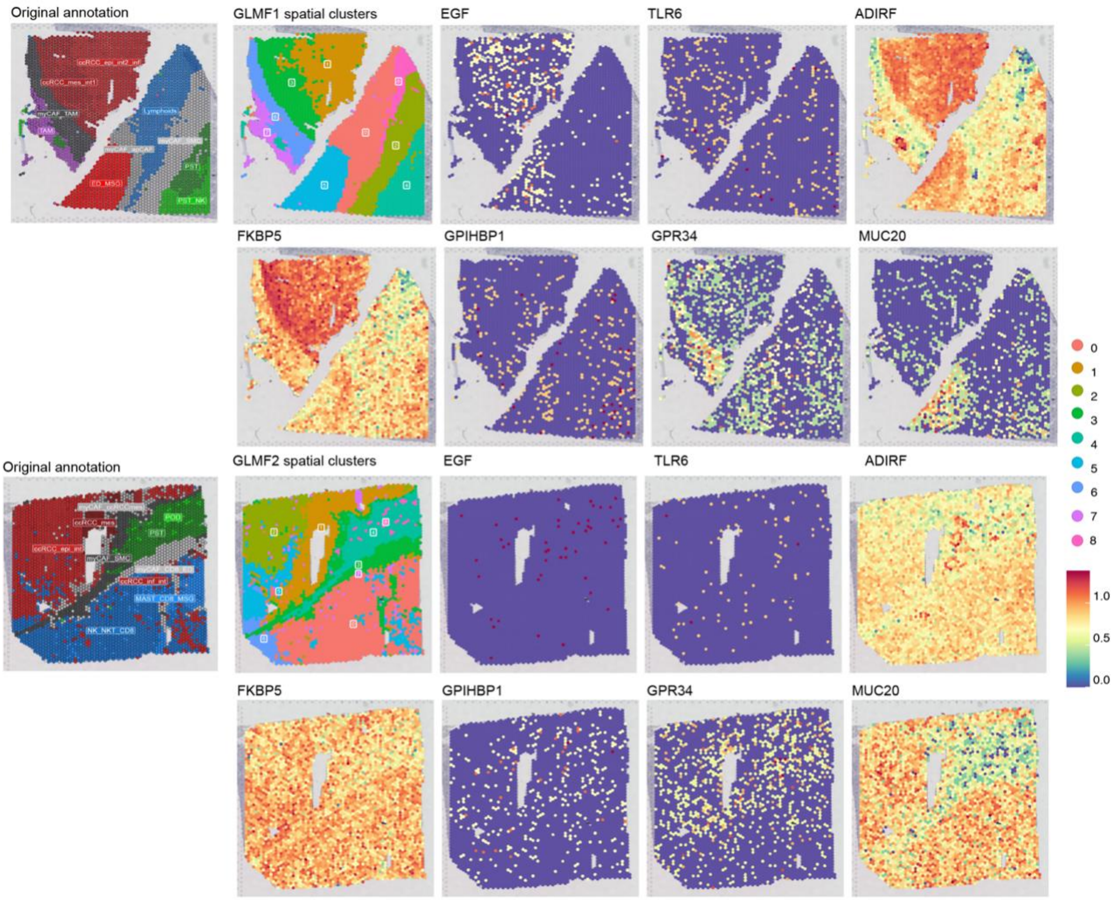

Fig.S4 Spatial plot showing the expression of nine key features.

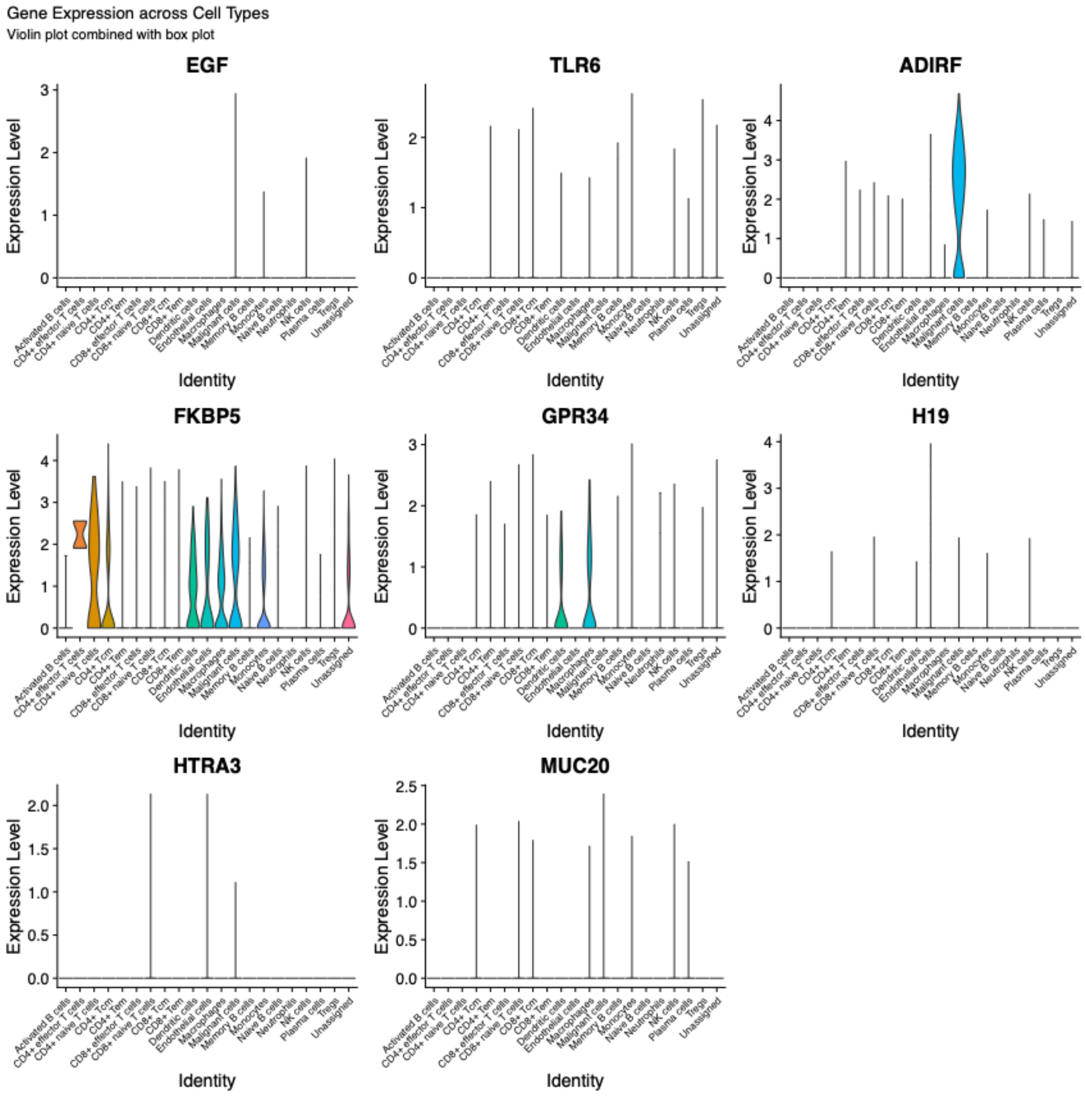

Fig. S5 Violin plot visualization of key gene expression across cell types.

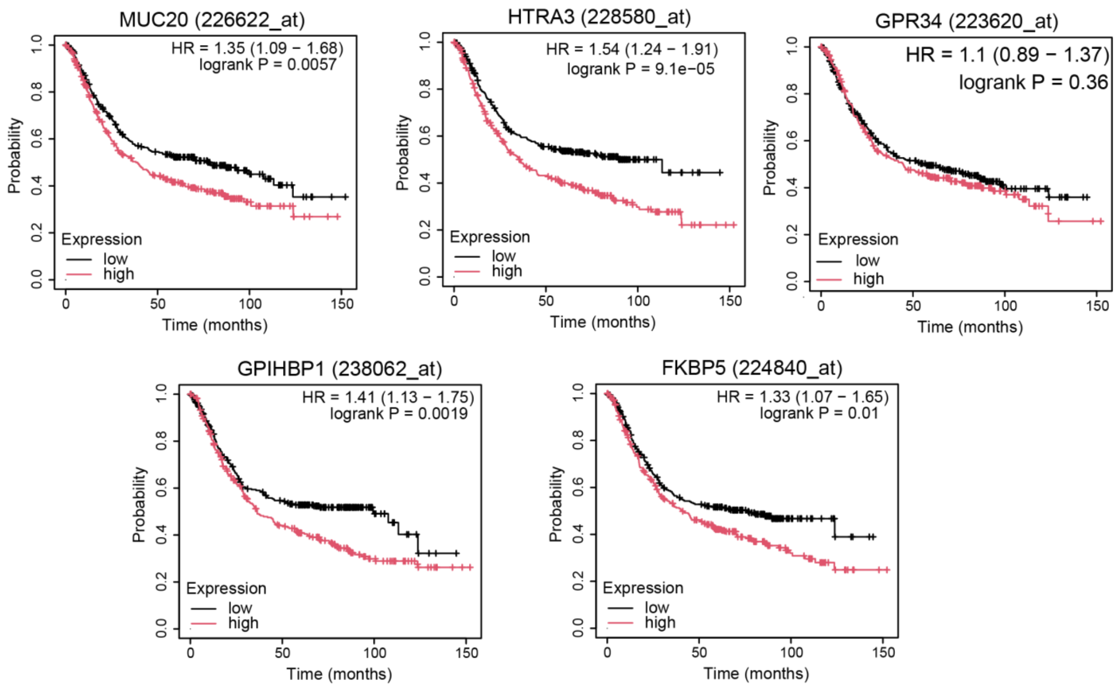

Fig. S6 The Kaplan-Meier survival curves based on key genes.

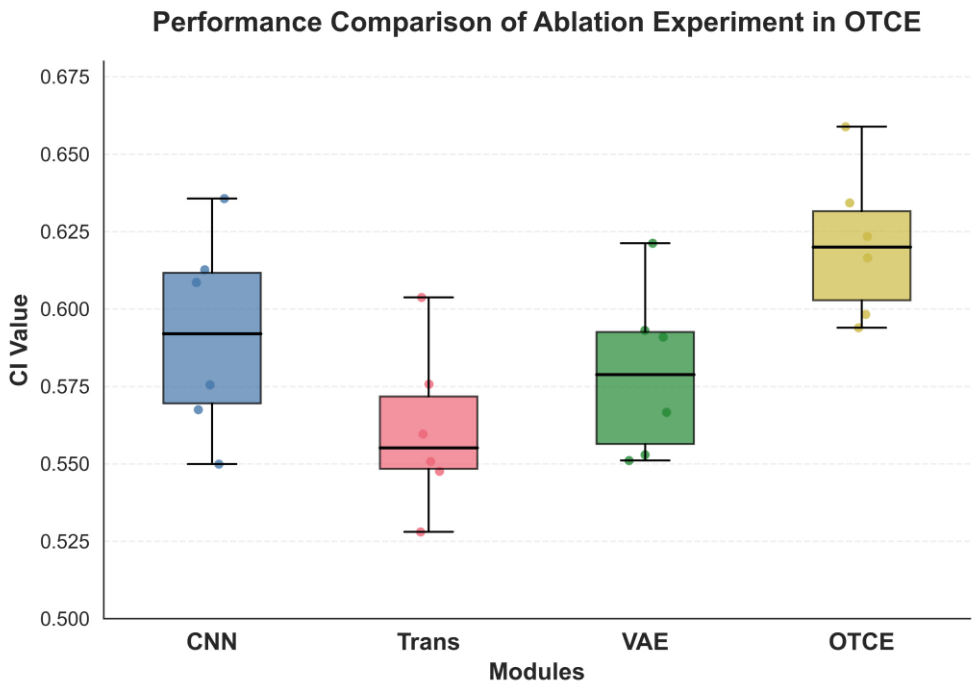

Fig. S7 Performance comparison of ablation experiment in 6 cancers by OTCE.

Table S1. Methods comparisons by C-index values achieved on different cancers.

	RSF	PCA	XGB	GDNet	DNN	DCAP	Ctrans	OTCE
BRCA	0.613 (±0.077)	0.623 (±0.062)	0.595 (±0.059)	0.680 (±0.060)	0.637 (±0.087)	0.649 (±0.108)	0.669 (±0.156)	0.624 (±0.087)
CESC	0.613 (±0.077)	0.581 (±0.055)	0.587 (±0.075)	0.603 (±0.042)	0.626 (±0.082)	0.639 (±0.089)	0.598 (±0.092)	0.614 (±0.068)
COAD	0.548 (±0.079)	0.499 (±0.013)	0.554 (±0.109)	0.620 (±0.053)	0.573 (±0.134)	0.598 (±0.017)	0.614 (±0.089)	0.659 (±0.103)
ESCA	0.547 (±0.103)	0.527 (±0.052)	0.573 (±0.080)	0.624 (±0.143)	0.539 (±0.050)	0.573 (±0.093)	0.609 (±0.156)	0.617 (±0.064)
KIRC	0.540 (±0.121)	0.524 (±0.057)	0.504 (±0.019)	0.600 (±0.092)	0.617 (±0.078)	0.569 (±0.069)	0.576 (±0.038)	0.634 (±0.026)
LUSC	0.519 (±0.037)	0.488 (±0.025)	0.523 (±0.075)	0.539 (±0.044)	0.522 (±0.030)	0.551 (±0.035)	0.564 (±0.086)	0.594 (±0.040)
Average	0.563	0.540	0.556	0.611	0.586	0.596	0.604	0.624
P-value[a]	4.00E-3	2.84E-3	1.81E-3	2.03E-1	2.65E-2	5.25E-2	9.26E-2	-

[a] The paired t-tests by comparisons with OTCE.

TableS2. Details about the model information and computational environment

S2.1 Transformer Module Configuration

The Transformer module was designed to capture long-range dependencies and global feature interactions from pseudo-image representations. The detailed configuration is as follows:

Embedding dimension: 256
Number of attention heads: 4
Transformer depth (number of encoder layers): 6
Patch embedding strategy:
Conv2D (kernel_size = 8, stride = 8)
Input channels = 3
Output embedding dimension = 256

This configuration allows the model to efficiently encode spatial feature relationships while maintaining computational tractability.

S2.2 Variational Autoencoder (VAE) Module

The VAE module was employed to capture latent feature representations and enhance model robustness through reconstruction regularization.
Latent dimension: 64
The VAE module introduces latent space regularization and improves feature compactness in the pseudo-image domain.

S2.3 CNN (Deep Convolutional Neural Network) Module

The CNN module focuses on local spatial feature extraction from the pseudo-images.
Layer-by-layer configuration:

Conv2d(3 → 64, kernel_size=3, padding=1)
MaxPool2d(2, 2)
Conv2d(64 → 128, kernel_size=3, padding=1)
MaxPool2d(2, 2)
Fully connected layer:
Linear(128 × 30 × 30 → 256)
The CNN output embedding dimension is aligned with the Transformer and VAE branches for subsequent attention-based feature fusion.

S2.4 Regularization Strategies
To improve generalization and prevent overfitting, the following regularization techniques were applied:
Weight decay: 1×10^{-5} (applied in optimizer)
These strategies help improve robustness under limited sample sizes.

S2.5 Software Environment
All experiments were conducted under the following software environment:
Operating System: Linux 5.4.0-144-generic (Ubuntu kernel)
Python: 3.12.3
NumPy: 1.26.4
PyTorch: 2.3.0 with CUDA 12.1
TensorFlow: 2.18.0
Pandas: 2.2.3

S2.6 Hardware Environment
CPU:Intel Xeon Silver 4214R @ 2.40 GHz
GPU: NVIDIA GeForce RTX 3080 Ti
